# A modular framework for biomedical concept recognition

**DOI:** 10.1186/1471-2105-14-281

**Published:** 2013-09-24

**Authors:** David Campos, Sérgio Matos, José Luís Oliveira

**Affiliations:** 1 IEETA/DETI, University of Aveiro, Campus Universitário de Santiago, 3810-193 Aveiro, Portugal

## Abstract

**Background:**

Concept recognition is an essential task in biomedical information extraction, presenting several complex and unsolved challenges. The development of such solutions is typically performed in an ad-hoc manner or using general information extraction frameworks, which are not optimized for the biomedical domain and normally require the integration of complex external libraries and/or the development of custom tools.

**Results:**

This article presents Neji, an open source framework optimized for biomedical concept recognition built around four key characteristics: modularity, scalability, speed, and usability. It integrates modules for biomedical natural language processing, such as sentence splitting, tokenization, lemmatization, part-of-speech tagging, chunking and dependency parsing. Concept recognition is provided through dictionary matching and machine learning with normalization methods. Neji also integrates an innovative concept tree implementation, supporting overlapped concept names and respective disambiguation techniques. The most popular input and output formats, namely Pubmed XML, IeXML, CoNLL and A1, are also supported. On top of the built-in functionalities, developers and researchers can implement new processing modules or pipelines, or use the provided command-line interface tool to build their own solutions, applying the most appropriate techniques to identify heterogeneous biomedical concepts. Neji was evaluated against three gold standard corpora with heterogeneous biomedical concepts (CRAFT, AnEM and NCBI disease corpus), achieving high performance results on named entity recognition (F1-measure for overlap matching: species 95%, cell 92%, cellular components 83%, gene and proteins 76%, chemicals 65%, biological processes and molecular functions 63%, disorders 85%, and anatomical entities 82%) and on entity normalization (F1-measure for overlap name matching and correct identifier included in the returned list of identifiers: species 88%, cell 71%, cellular components 72%, gene and proteins 64%, chemicals 53%, and biological processes and molecular functions 40%). Neji provides fast and multi-threaded data processing, annotating up to 1200 sentences/second when using dictionary-based concept identification.

**Conclusions:**

Considering the provided features and underlying characteristics, we believe that Neji is an important contribution to the biomedical community, streamlining the development of complex concept recognition solutions. Neji is freely available at http://bioinformatics.ua.pt/neji.

## Background

A growing amount of biomedical data is continuously being produced, resulting largely from the widespread application of high-throughput techniques, such as gene and protein analysis. This growth is accompanied by a corresponding increase of textual information, in the form of articles, books and technical reports. In order to organize and manage these data, several manual curation efforts have been set up to identify entities (e.g., genes and proteins) and their interactions (e.g., protein-protein). The extracted information is then stored in structured knowledge resources, such as MEDLINE and Swiss-Prot. However, manual annotation of large quantities of data is a very demanding and expensive task, being difficult to keep these databases up-to-date. These factors have naturally led to increasing interest in the application of text mining (TM) systems to help perform those tasks. One major focus has been on Named Entity Recognition (NER), a crucial initial step in information extraction, aimed at identifying chunks of text that refer to specific entities of interest. However, biomedical entity names present various characteristics that hinder the identification of those mentions in scientific documents [1]:

● Many entity names are descriptive (e.g., “normal thymic epithelial cells”);

● Two or more entity names may share one head noun (e.g., “91 and 84 kDa proteins” refers to “91 kDa protein” and “84 kDa protein”);

● One entity name with several spelling forms (e.g., “N-acetylcysteine”, “N-acetyl-cysteine”, and “NAcetylCysteine”);

● Ambiguous abbreviations (e.g., “TCF” may refer to “T cell factor” or to “Tissue Culture Fluid”).

In an effort to deal with these challenges, several NER systems have been developed for the biomedical domain, using different approaches and techniques that can generally be categorized as being based on rules, dictionaries or machine learning (ML). Each approach has different resource requirements and deals differently with the linguistic variability that resulted from the lack of naming standards and the introduction of idiosyncratic names by the scientific community [2]. In general, ML-based solutions are better adapted to deal with strong variability and highly dynamic vocabularies, such as in gene and protein names. However, this approach does not directly provide identifiers for the recognized names. Thus, normalization must be performed in an extra step in order to relate each name with concept identifiers from curated databases or ontologies. In this case, a concept corresponds to a biological entity present on curated and specialized resources used to represent and map current knowledge. On the other hand, dictionary-based approaches are appropriate to deal with precisely defined vocabularies of names (e.g., diseases and species). This approach requires the construction of a unique resource containing most of the identifiers and names of a specific semantic type. However, this presents various challenges, since the necessary information is usually spread over dozens of data sources and unique identifiers are specified on a per-resource basis, which hinders mapping identifiers between heterogeneous databases. Moreover, the same name may refer to different concepts, depending on the context in which it occurs. For instance, “NF1” can refer to a disease (“Neurofibromatosis Type 1”) or to a protein (“Neurofibromin 1”).

The development of NER and normalization solutions requires the application of multiple techniques, which can be conceptualized as a simple processing pipeline [2]:

● Input: interpret and filter input data to be processed;

● Pre-processing: process the input data in order to simplify the recognition process;

● Recognition: identify entity mentions from pre-processed data;

● Post-processing: refine generated annotations, solving problems of the recognition process or extending recognized names;

● Output: generate a structured output with the final annotations.

Each step of the processing pipeline may involve the implementation of various methods to fulfill the associated requirements. Due to the specificities of the biomedical domain, methods developed for common English may not provide the best outcomes when used on scientific documents. For instance, in [3] the authors analyzed the application of various tokenizers, concluding that most solutions are too simplistic for real-life biomedical applications. Thus, it is important to develop and use methods optimized to deal with the special linguistic characteristics of biomedical terms.

**Figure 1:**
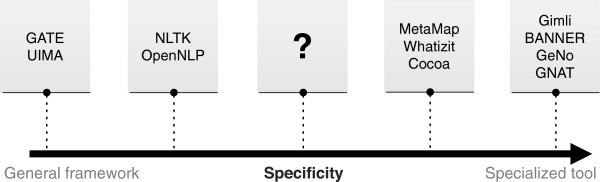
Spectrum of existing solutions for biomedical concept recognition according to their specificity.

Based on the general processing pipeline and considering the requirements of the biomedical domain, various solutions were implemented and used to support and streamline the development of complex biomedical IE solutions. Figure 1 presents the spectrum of frameworks and tools considering their relative specificity for this domain. The edges of the spectrum represent two contrasting types of solutions:

● General frameworks (left edge), which support the development of IE solutions with a pre-defined and general processing pipeline;

● Specialized tools (right edge), centered on the recognition of specific biomedical entity types and providing end-user features.

UIMA [4] and GATE [5] are examples of frameworks that provide a general solution to support the development of complex IE systems, being independent of the target domain. Such goal is achieved by providing a flexible processing pipeline based on a modular infrastructure, enabling problem decomposition and consequent re-utilization of modules. Besides the flexibility and re-usage advantages, such solutions also provide a strong infrastructure, such as cluster processing support for large amounts of data. However, due to the high level of abstraction, the development of new solutions may not be as straightforward as expected, requiring some time to correctly understand and have full control over the frameworks’ features. Moreover, neither framework provides default modules optimized for the biomedical domain, which are provided by third parties, such as U-Compare [6] and JCoRe [7] for UIMA. Nevertheless, most of those modules are only available through web-services, which is an optimal solution for small experiments but not compatible with large scale and batch processing applications. Additionally, users must be careful when using modules from different providers in a single pipeline, since the application of different techniques (e.g., tokenization and sentence splitting) among different modules may considerably degrade performance results.

Toolkits such as NLTK [8] and OpenNLP [9], on the other hand, are not focused on providing a text processing pipeline, offering instead a multitude of implemented methods that developers can use and combine to build their own pipelines. Various features of OpenNLP are also available as modules for UIMA, which may simplify the creation of such pipelines. However, these solutions do not provide modules optimized for the biomedical domain. Instead, they allow training new modules focused on different goals and domains.

On the opposite edge of the spectrum are specialized NER and normalization tools, whose development was greatly promoted through the organization of challenges such as BioCreative [10-12] and JNLPBA [13]. Dozens of new solutions emerged using the resources provided by these challenges, which allowed a fair and fast comparison of divergent techniques. Gimli [14] and BANNER [15] are examples of NER solutions, and GeNo [16] and GNAT [17] are examples of NER and normalization tools. However, the resources provided by those challenges are too specific and focused on the recognition of particular entity types (e.g., gene and protein), generating highly optimized solutions that provide high performance results on tested corpora. NER solutions are typically open-source and publicly available as runnable applications, enabling re-usage of already implemented modules and fast development of new recognition systems. However, there is no explicit processing pipeline and such solutions are not flexible, limiting the addition or removal of processing modules. On the other hand, normalization solutions are mostly not open-source, providing only web-services for remote usage, which is limited for batch processing.

There are also solutions focused on providing annotation of heterogeneous biomedical concepts. For instance, Whatizit [18], Cocoa [19] and NCBO Annotator [20] provide annotations of species, genes and proteins, and disorders, among others concepts. However, since they are provided as web-services, batch processing is limited and desirable functionalities, such as the possibility to configure annotation characteristics or to extend the provided features, are not available. MetaMap [21] is another tool that provides annotation of heterogeneous concepts, using the UMLS Methathesaurus and a set of rules for extracting text chunks and scoring them as candidates for concept names. Matching is performed considering lexical and syntactic rules, generating names variants to cover as much variability as possible. However, such approach makes MetaMap relatively slow, not being appropriate for real-time use. For instance, it may take several hours to process complex sentences, generating many hundreds of thousands of potential mappings [22]. On the other hand, the variability introduced also increases ambiguity, which is a complex problem to solve. Moreover, since it is provided as an end-user tool, it is also limited in terms of configurability and extensibility.

Considering the current frameworks and tools for the biomedical domain, we believe there is a lack of solutions that combine the advantages of the two edges of the spectrum: modularity, speed, usability and domain optimization. This document presents Neji, an open source framework for biomedical concept recognition that provides an automated and flexible processing pipeline that includes built-in methods optimized for the target domain. It supports the application of both machine learning and dictionary-based approaches, automatically combining generated annotations and supporting concept ambiguity. Neji also supports known input and output formats, with easy development of new pipelines and modules.

We believe that Neji is a positive contribution for the biomedical community, by simplifying the development of complex concept recognition solutions and taking advantage of the most advanced and appropriate methods in an integrated environment focused on fast and high-performance results. As a result, we believe that Neji may enhance text mining and knowledge discovery processes, helping researchers in the annotation of millions of documents with dozens of biomedical concepts, in order to infer new biomedical relations and concepts.

In the next section, we give a detailed description of Neji’s modular architecture, presenting the core infrastructure, the included modules and its usability. Afterwards, Neji is evaluated in term of concept annotation accuracy and speed. In the end, we discuss the main advantages and applications of Neji.

## Implementation

The design and implementation of Neji was focused on four crucial characteristics: modularity, scalability, speed and usability. In order to achieve modularity, every processing task is performed by an independent module, which can be executed ad-hoc or integrated in a processing pipeline. Nonetheless, each module has its own input and output specifications. Regarding scalability, the solution should be able to support simultaneous application of dozens of dictionaries and machine-learning models for concept recognition, while at the same time processing large data sets (i.e., millions of abstracts). One of the key features to deal with large data sets and considerably improve processing times is concurrent processing, allowing different CPU cores to process several documents at the same time. Additionally, it is also fundamental to take processing speed into consideration when choosing libraries and techniques to perform the different steps. Finally, developers and researchers should be able to easily use pre-defined pipelines, implement custom pipelines with provided modules and/or implement new modules respecting previously specified interfaces. Moreover, typical processing modules, such as sentence splitting and tokenization, should be part of the framework and available for direct use and/or extension.

A framework with such characteristics should be an added value for the biomedical community, allowing any user to easily develop custom and complex solutions and use them according to their specific goals. Additionally, advanced users do not need to deal with various independent tools and libraries, allowing them more time to dedicate to their real goals.

### Infrastructure

**Figure 2:**
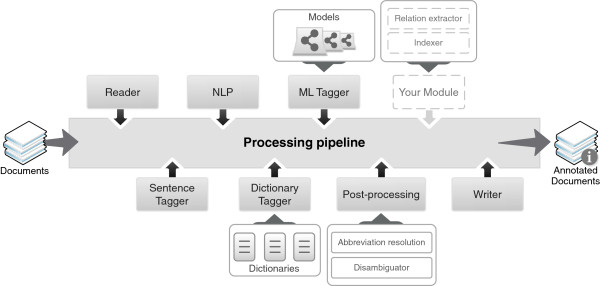
Illustration of the processing pipeline and modular architecture of Neji.

**Figure 3:**
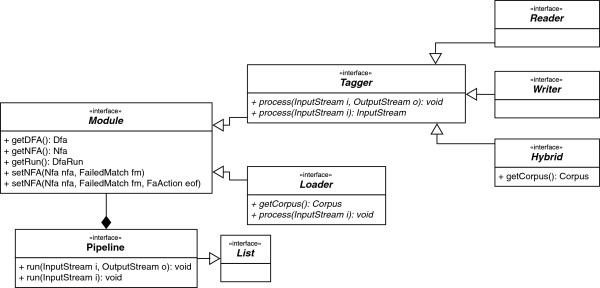
Interface diagram to model implementation of pipelines and respective modules.

The core component of Neji is the pipeline, which allows users to submit various modules for execution following a FIFO (First In, First Out) strategy. Thus, a pipeline is a list of modules that are executed sequentially, considering specific goals and target chunks of text. Figure 2 illustrates the idea of this modular and flexible architecture. Each module is implemented as a custom Deterministic Finite Automaton (DFA), with specific matching rules and actions. We used the hierarchical text processing features of Monq.jfa [23] to support the pipeline infrastructure and module execution (Figure 3). When a pipeline is executed, the input documents are the input of the first module, and the output of the first module is the input of the second module and so on, until the last module provides the output to a storage resource specified by the user. Since different tasks have different requirements, different types of modules are defined:

● Tagger: processes the input data and reflects the changes in the same data. For instance, when performing sentence splitting, inline annotations can be provided to reveal the obtained sentences;

● Loader: loads information present on the input data into memory. For instance, if inline biomedical name annotations are present in the input text, a loader can be used to load such annotations into memory;

● Hybrid: processes input data and store the results in internal memory. Inline annotations can also be provided as output. For instance, when performing sentence splitting, it should be useful to provide inline annotations of the sentences and load them into memory. Obviously, a tagger and a loader can be used instead, but some processing time is wasted in reading the annotations from the tagger to the loader;

● Reader: a Tagger that is used to collect data of interest from the input resource;

● Writer: a Tagger that is used to generate output data to a specific resource.

In order to support default and basic behaviors, Neji already provides implementations of the various components, namely tagger, loader, reader, writer, hybrid and pipeline. Such architecture allows developers to easily build custom module types or pipelines.

**Figure 4:**
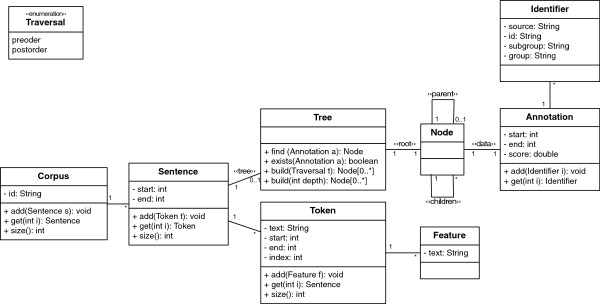
Overview of the internal data structure to support processed data.

**Figure 5:**
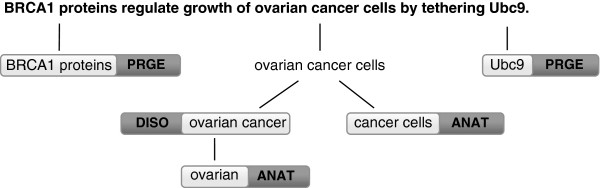
**Illustration of implemented concept tree.** Such structure automatically supports nested and intersected concepts, clearly exposing ambiguity problems (PRGE: Proteins and genes; DISO: Disorders; and ANAT: Anatomy).

Since Neji is a framework focused on biomedical concept recognition, it also defines and provides a flexible and complete data structure to represent a corpus. Thus, developers do not need to specify their own internal data structures, and they can easily extend the provided data representation. Figure 4 illustrates the final internal data representation of a corpus with sentences and respective annotations. Moreover, since Neji supports automatic annotation of heterogeneous biomedical concepts, in which the existence of nested and/or intersected annotations is common, it is important to integrate a data structure that suits such characteristics in the best and most automated way as possible. A tree of annotations is the data structure that better fulfills such requirements, presenting various advantages over typical approaches (e.g., list of annotations): *a*) structured annotations provide enhanced information, since nested and intersected annotations and their respective identifiers are provided; *b*) the levels of the tree are directly associated with the detail of annotations, the deeper the level the more deeply an annotation is nested and/or intersected in others; *c*) the consistency of the tree and of the respective annotations can be maintained through automatic algorithms; *d*) ambiguity problems are clear; and *e*) filtering annotations can be as simple as pruning the tree. As illustrated in Figure 5, each sentence includes a tree of annotations. In order to facilitate the use and management of these trees, as well as for maintaining the consistency of the annotations, the following methods are provided:

● Sorted insert: when an annotation is added to the tree, it is automatically put in place, maintaining the tree consistency;

● Sorted delete: when an annotation is removed from the tree, all other annotations are put in place in order to keep tree consistency;

● Traversal: obtain a list of ordered annotations following typical tree traversal techniques: by level, pre and post-order;

Since an annotation without concept identifiers is less informative, it is important to provide an infrastructure that allows each annotation to contain various identifiers. Moreover, each identifier should provide complete information regarding its original source and concept type. Thus, the following quadruple composes each identifier: source (original resource that contains the name and respective identifier); identifier (unique identifier of the concept in the previously specified resource); group (semantic group of the concept); and sub-group (semantic type of the concept).

### Modules

With the proposed infrastructure, the conditions to build the required modules for text processing and concept recognition are now met. The presentation of modules follow the processing pipeline previously presented and illustrated in Figure 2.

#### Readers

A reader module is used to interpret input data, in order to collect the relevant data and convert it into a format that is readable by the following modules. Instead of obtaining the relevant data and storing it into memory, we decided to use a tagger to mark the original input text with regions of interest (ROI) tags (“<roi > text</roi>”). Thus, the following modules only have to match the ROI tags and process the contained text. Two different reader modules are already provided, allowing to process XML and raw text. The XML module allows developers to specify the tags of interest. For instance, considering the Pubmed XML format, if only titles and abstracts have to be processed, only the content of the tags “ArticleTitle” and “AbstractText” are of interest. On the other hand, the raw reader considers that all the input text is of interest to be processed.

#### Natural language processing

After obtaining the texts of interest, the next fundamental step is to perform sentence splitting, since a sentence is the basic unit of logical thought. This phase presents various complex challenges due to the specific characteristics of scientific biomedical texts [24,25]. Thus, we integrated a module to perform sentence splitting taking advantage of the Lingpipe [26] library, which contains a sentence splitting model trained on biomedical corpora and presents high-performance results [27]. Natural Language Processing (NLP) tasks are performed using GDep [28], a dependency parser for the biomedical domain built on top of the GENIA tagger, which performs tokenization, lemmatization, part-of-speech (POS) tagging, chunking and NER. Since we are not interested in the named entities provided by the GENIA tagger, we removed the module and its dependencies. Moreover, we decided to make the tokenizer behavior more consistent, by breaking words containing the symbols “/”, “-” or “.” into multiple tokens, which improved results [14]. Because GDep combines all the tasks in order to perform dependency parsing, we decoupled the various processing tasks, obviously respecting all task dependencies and resources (tokenization < POS < lemmatization < chunking < dependency parsing). Thus, for each task, only the required resources (e.g., models) are loaded. For instance, if one needs the pipeline just for dictionary matching, only the tokenization plugin will be loaded and executed. On the other hand, when dependency parsing is required, all the processing tasks are performed and respective information provided. For instance, if a machine-learning model uses tokens, POS and lemmas as features, but not chunks or parsing features, these two tasks are not performed, making the process considerably faster.

#### Concept recognition

As stated before, distinct biomedical concepts require distinct approaches in order to achieve more accurate recognition. Thus, Neji provides concept recognition using both dictionary and machine learning-based approaches. Dictionary matching is offered using a modified version [29] of the dk.brics.automaton [30] library, which provides efficient regular expression matching with Deterministic Finite Automatons (DFAs). In a simplistic way, DFAs are finite state machines that accept or reject finite strings of symbols. Thus, a DFA transits from one state to another, depending on the sequence of input symbols, and a string is accepted if its parsing finishes in a state marked as final. Considering that each input string of symbols is a name from the dictionary, one can build a DFA to match all names in a dictionary. Additionally, each regular expression representing a name from the dictionary is associated with a specific identifier, enabling concept recognition. Such approach supports both exact and approximate matching, and performs the recognition of named entities in *O*(*n*) time, where *n* is the size of the document. Since a large amount of false positives may be generated using approximate matching, and considering that we are dealing with a general biomedical solution, we decided to use case insensitive exact matching. Orthographic variants of names can be generated and provided in the dictionary. Even so, it is necessary to pay special attention to terms that are common English words. Thus, a list of non-informative words for the biomedical domain [31] is ignored during the matching process. Similarly, biomedical names with two characters or fewer are also discarded. However, such a strategy may mean that acronyms of known entity mentions would be missed, which can be overcome by a post-processing module for acronym resolution.

Dictionaries are provided in TSV (tab-separated values) format with two fields: identifier and list of names. Identifiers should follow the format “source:identifier:type:group” and their respective names must be concatenated with a pipe (“|”). To allow easy configuration and support dozens of dictionaries, files must be provided in a folder with an additional priority file, which contains the file names of the dictionaries (one per line) and defines the priority to be used if a disambiguation method is applied. This simple strategy enables fast, easy and flexible configuration of dictionaries.

In order to optimize the concept recognition results, some directives are followed when applying dictionary matching, assuming that a different dictionary file is used for defining concepts in each semantic group or type:

● Considering one dictionary (i.e. same semantic group/type), only the entry with the largest span is matched;

● If two entries with the same text exist, in the same or in different dictionaries, both entries are matched and both identifiers are provided;

The support of machine learning-based solutions is provided through Gimli, which uses the Conditional Random Fields (CRFs) implementation from MALLET [32] to recognize various biomedical entity types, and provides high-performance results in two well-known corpora: GENETAG [33] and JNLPBA [13]. It also provides a complete set of basic and complex features, serving as a good starting point to develop NER solutions for the biomedical domain. Thus, various CRF models trained for Gimli can also be used in Neji, each one focused on a different biomedical concept type. Gimli already provides models for the recognition of gene and protein names, trained on GENETAG, and for the recognition of gene and protein, DNA, RNA, cell type and cell line names, trained on JNLPBA. Nonetheless, developers can also use Gimli to easily train new models on different corpora and/or focused on different entity types. However, Gimli only performs NER, not establishing a relation between chunks of text and unique identifiers from curated databases. Thus, we developed a simple and general normalization algorithm based on prioritized dictionaries. Following this algorithm, if an identifier is found in the first dictionary, the match is complete and the algorithm finishes. If no match is found in the first dictionary, the second one is used to find a match, and so on. In the end, if no matches are found in the provided dictionaries, the developers can choose to keep or discard the annotation. This configuration works well if the first dictionary contains a list of preferred names, and the remaining contain synonyms for each identifier. Using this setting, a mention to “TRAF2” would be matched in the first dictionary, since this is the preferred symbol for the gene associated with the protein with Uniprot accession Q12933, and the matching process would stop. Additionally, “TRAF2” is also a synonym for the gene “TANK” (Uniprot accession Q92844), but since this is defined in a dictionary with lower priority the match would not occur. Moreover, this strategy also provides flexibility to users, which only have to generate the various orthographic variants and prioritize them in the dictionaries. Regarding the matching approach, if a partial match of the annotation is found in the dictionary, it is accepted as a valid identifier for the complete chunk of text. For instance, if only “BRCA1” is present in the dictionary, and the annotation “BRCA1 gene” is provided, the identifier of “BRCA1” is associated with the annotation. Conversely, if “BRCA1 gene” is in the dictionary and “BRCA1” is found in the text, a match is not obtained since “extra” tokens are only considered in the textual mention and not in the dictionary entries. ML models are provided to Neji following a similar approach of dictionaries, where a properties file defines the characteristics of each model.

#### Post-processing

Neji is also able to integrate post-processing modules, in order to optimize previously generated information. By default, an abbreviation resolution module is provided, in order to extend existing concepts. Thus, we adapted a simple but effective abbreviation definition recognizer [34], which is based on a set of pattern-matching rules to identify abbreviations and their full forms. In this way, we are able to extract both short and long forms of each abbreviation in text. If one of the forms is already provided as a concept, the other one is added as a new concept with the identifiers of the existing one. Additionally, any further occurrences of that entity are also automatically annotated.

Depending on user requirements, it may be useful to filter concept annotations following pre-defined rules. Thus, Neji provides the ability to remove annotations from the concept tree based on three simple disambiguation strategies:

● By depth: remove annotations from the concept tree that are deeper than a specified depth;

● Nested same group: remove concept annotations that are nested on annotations of the same semantic group and with a larger span;

● By priority: remove nested and intersected concept annotations following a prioritized list.

#### Writers

**Figure 6:**
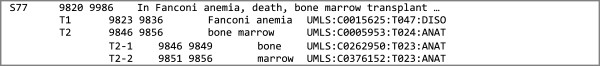
Example of the Neji output format.

Writers are used to store the recognized concepts in external resources, such as files and databases. If the user does not want to provide the result into an external resource, the corpus is programmatically available. Neji supports various well-known inline and standoff formats used in the biomedical domain, such as IeXML [35], A1 [36], CoNLL [37] and JSON [38]. Overall, identifiers are provided following the format “source:identifier:type:group”, and using a pipe (“|”) to concatenate various identifiers for a single annotation. IeXML is an inline annotation format based on XML tags, supporting two levels of detail, i.e. only one annotation nested or intersected in another. Moreover, various identifiers can be provided using IeXML. Both CoNLL and A1 support ambiguous and intersected concept annotations. However, complex identifiers are not supported in CoNLL, thus only the semantic group is provided. The output of the A1 format can be used with brat [39], in order to visualize and edit the generated annotations. Finally, the JSON format provides all the information contained in the tree, together with the sentence and respective character positions. We also provide our own format, in order to overcome some limitations of other formats regarding nested/intersected annotations and multiple identifiers. It can be seen as an alternative to JSON, being more readable and understandable by humans. Figure 6 presents an example of the Neji output generated for a sentence. As we can see, each sentence has its own identifier, start and end character positions, and respective text. Regarding annotations, an indentation-based approach is used to reflect the tree hierarchy, accompanied with the respective term identifier, start and end character positions, and associated text and identifiers.

### Parallel processing

In order to simplify the use of the various modules and required resources, we developed a method to manage these resources, which we call Context. It automatically loads the resources that are required to run a specific pipeline. Thus, researchers do not need to deal with repetitive and time consuming tasks such as loading dictionaries, ML models, parsers and sentence splitters. Additionally, we also provide parallel processing of documents through multi-threading support. To accomplish this, the libraries and respective dependencies used were adapted to allow multi-threaded execution, solving some limitations with MALLET and GDep. The Context also supports multi-threading, by automatically generating the required duplicate resources when necessary. For instance, concurrent annotation of documents using one ML model is not possible, requiring one instance of the ML model for each thread. In order to apply parallel processing, each pipeline must be implemented in a Processor, which is a runnable pipeline with context and input and output resources specification. Base implementation of a Processor is already provided, which simplifies the development of alternative runnable pipelines. A Batch is also provided, which performs concurrent processing of input resources using a specific Processor and Context. Considering the typical use case scenario of parallel processing in the biomedical domain, i.e., process files in an input folder and provide the results to an output folder, we developed a Batch executor to make the applicability of parallel processing easier. The Batch automatically generates the required Processor threads to process specific files in a folder. Custom arguments for the processors can be also provided, which takes advantage of Java reflection.

### Usage

In order to make the annotation process as simple as possible in typical use cases, Neji integrates a simple but powerful Command Line Interface (CLI) tool, which is flexible and provides a complete set of features:

● Annotate using dictionaries and/or machine-learning models with respective normalization dictionaries;

● Various input and output formats. When the XML input format is used, the XML tags should be indicated;

● Parsing level customization. By default, Neji automatically finds the appropriate parsing level considering the ML model characteristics;

● Number of threads customization;

● Wildcard input filter to properly indicate the files to process;

● Support for compressed and uncompressed files.

The features provided by the CLI tool allow annotating a corpus using a simple bash command, such as:

./neji.sh -i input/ -if XML -o output/ -of XML -x AbstractText,ArticleTitle -d resources/dictionaries/ -m resources/models/ -c -t 6

**Figure 7:**
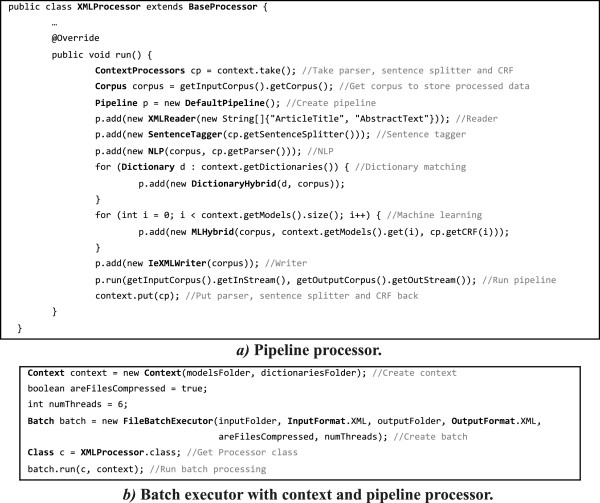
Java code snippets to create a runnable processing pipeline and use it in a batch executor with context.

In this example, Neji uses six threads to annotate the compressed XML documents in the input folder with the specified dictionaries and machine-learning models, providing the resulting XML documents to the output folder. Note that only the text inside the specified tags is annotated. If users do not want to use the provided CLI, it is also straightforward to develop a processor and process the documents using the batch helper. First, a processor taking advantage of the pipeline features must be implemented. Figure 7:a presents the construction of a complete pipeline processor that produces the same results as the previous bash command, considering a specific context, input and output documents provided in the constructor. Afterwards, this pipeline processor must be used to perform batch processing of documents. Figure 7:b shows how a context is created considering input models and dictionaries folders, and how a batch is created for specific input and output folders. Finally, the batch is executed considering the provided context and all documents are annotated. Complete and detailed documentation on how to use the CLI tool, build custom processors, and build processing modules is provided in the Neji’s web page.

## Results

To provide general feedback regarding Neji’s reliability as a framework, it is fundamental to evaluate its behavior on real life problems. Thus, we believe that such framework should be evaluated considering two key characteristics:

● Concept annotation: how is the quality of the produced concept annotations?

● Speed: how long it takes to process a specific amount of documents?

Accordingly, we collected manually annotated corpora, dictionaries and ML models to take advantage of Neji, and compared the achieved performance results with existing solutions.

### Corpora

Our primary analysis was centered on the CRAFT corpus [40], one of the largest publicly available gold standard corpora, which is focused on multiple biomedical concept types with heterogeneous characteristics. The initial release contains a set of 67 full-text articles (more than 21 thousand sentences) manually annotated by domain experts, focused on nine biomedical ontologies and terminological resources: Chemical Entities of Biological Interest (ChEBI); Cell Ontology; Entrez Gene; Gene Ontology (biological process, cellular component, and molecular function); NCBI Taxonomy; Protein Ontology and Sequence Ontology. Overall, it contains almost 100 thousand annotations. However, CRAFT does not include anatomical and disorder concepts, which we believe are fundamental to cover the general biomedical concept spectrum. Thus, we decided to use two other corpora for concept annotation evaluation. The AnEM [41] corpus is focused on anatomical entities, using a fine-grained classification system based on the Common Anatomy Reference Ontology (CARO). The annotated concepts are precisely divided into eleven anatomical class labels, such as “Organ”, “Tissue”, “Cell” and “Organism substance”. This corpus is based on 250 abstracts and 250 full-text extracts (article sections) randomly selected from PubMed and from PubMed Central (PMC), containing 3135 manually annotated concepts. For testing purposes, 100 abstracts and 100 full-text extracts are provided, summing together 1879 annotated concepts. Finally, the third was the NCBI disease corpus [42], produced by expert annotators using the Unified Medical Language System (UMLS) as reference resource and containing disease concepts classified into four class labels: Specific Disease, Disease Class, Composite Mention and Modifier. It contains 793 abstracts (6651 sentences) from PubMed with 6900 disease mentions. For testing purposes, 100 abstracts with 961 mentions are provided. In the end, we used the 67 full-text articles of the CRAFT corpus, and the test parts of both AnEM and NCBI corpora, in order to allow direct and fair comparison.

### Resources

Considering the three corpora, we collected the ML models and/or dictionaries described below to recognize biomedical concepts of each type. Resources for the ‘Disorders’ and ‘Anatomy’ types were used for annotating the NCBI disease and AnEM corpus, respectively, and the remaining were considered for the CRAFT corpus.

● Genes and proteins: due to the variability of gene and protein names, their recognition was performed using a ML model trained on GENETAG. It applies a complete and complex set of features, namely lemmas, POS, chunking, orthographic, local context (windows) and morphological features. LexEBI [43], which contains a filtered version of BioThesaurus [44], the most complete resource of gene and protein names, is used to perform normalization. The dictionary was further filtered to only include gene and protein names for 21 of the most commonly studied species ^a^. Two different dictionaries were generated: the first with preferred names and the second with synonyms for each identifier. Additionally, for each dictionary a set of orthographic and semantic variants was generated using the Lexical Variants Generation (LVG) tool [45], namely: *a*) derivational, uninflected and inflectional name variants; *b*) strip ambiguous words, punctuation symbols and plural suffixes; *c*) known synonyms and variants from biomedical databases; and *d*) invert names around commas. In the end, four dictionaries were used with the following matching priority: *1*) preferred terms; *2*) synonyms; *3*) preferred terms with variants; and *4*) synonyms with variants; A simple filtering of gene and protein identifiers was also applied as a post-processing step, by discarding identifiers associated with species that are not named in the document. Thus, if identifiers for human and mouse proteins are provided for a recognized protein name and mice are not referred in the document, the identifier for the mouse protein is removed from the protein annotation.

● Chemicals: a dictionary of chemical names was built using the ChEBI database of molecular entities [46];

● Species: the dictionary provided by LINNAEUS [29] was extended by adding the entries from the NCBI Taxonomy assigned to taxonomical ranks above “species”, that is, from “genus” to “domain”. For each entry, we included the names from NCBI as well as the synonyms obtained from the corresponding concept in the Unified Medical Language System (UMLS) Metathesaurus [45]. Furthermore, less specific names for species that also appeared as names in higher taxonomical levels, such as the genera “rat” or “mouse”, were filtered and kept only at the highest level, in order to approximate the annotation guidelines used in the CRAFT corpus;

● Cells: cell names were compiled from the “Cell” and “Cell Component” semantic types in the UMLS Metathesaurus;

● Cellular Component, Biological Process and Molecular Function: terms for these concept types were obtained from the corresponding sub-ontologies of the Gene Ontology (GO) [47], and expanded with synonyms from the corresponding concepts in the UMLS Metathesaurus. Additionally, UMLS concepts assigned to the UMLS semantic types “Physiologic Function”, “Organism Function”, “Organ or Tissue Function”, “Cell Function”, “Molecular Function” and “Genetic Function” were also included since they identify concepts closely related to biological processes and molecular functions, even if they are not directly mapped to GO terms;

● Disorders: names and synonyms for abnormalities, dysfunctions, symptoms and diseases were extracted from the Metathesaurus. We considered the following UMLS semantic types assigned to the “Disorders” semantic group: “Acquired Abnormality”, “Anatomical Abnormality”, “Congenital Abnormality”, “Disease or Syndrome”, “Mental or Behavioral Dysfunction”, “Neoplastic Process”, “Pathologic Function” and “Sign or Symptom”;

● Anatomy: anatomical entities were extracted from the Metathesaurus, considering the following semantic types grouped under the “Anatomy” semantic group: “Anatomical Structure”, “Body Location or Region”, “Body Part”, “Organ, or Organ Component”, “Body Space or Junction”, “Body Substance”, “Body System”, “Cell”, “Cell Component”, “Embryonic Structure” and “Tissue”. The semantic type “Fully Formed Anatomical Structure” was not included, as it contains only a few very general terms, such as “total body” or “whole body structures”. The terms from the “Cellular Component” sub-ontology in GO were also included. Additionally, we included the terms from the “Neoplastic Process” semantic type since this most closely matches the “Pathological Formation” annotation type included in the AnEM corpus.

As a filtering step to eliminate inconsistent names and names that would generate a large number of false positives, we rejected names with one or two characters, names starting with a word from a strict list of stopwords (e.g. “very long chain fatty acid metabolic process”, “the cell”), and also any single word name if that word was included in a broader list of stopwords generated from the list of most frequent words in MEDLINE. Some relevant terms that occur very frequently in MEDLINE, such as general names of diseases (e.g. “cancer”, “diabetes”), Gene Ontology terms (e.g. “expression”, “transcription”) and species (e.g. “human”, “Saccharomyces”), were removed from this stopword list to allow identifying them in texts.

As can be seen, different resources are used for each of the considered concepts in order to provide the best and most complete results as possible, an approach greatly simplified by Neji's modular pipeline. In the end, our dictionaries contain almost 1 million concept identifiers with 7 million name variants.

### Concept annotation evaluation

Two different evaluation approaches were performed, in order to fully assess the quality of the provided concept names and identifiers:

● Named entities: evaluate the quality of the provided text mentions discarding the assigned identifiers;

● Normalization: evaluate the quality of the text mentions together with the assigned identifiers.

Regarding the evaluation of named entities, five matching techniques were considered:

● Exact: annotation is accepted if both left and right sides match with the gold standard annotation;

● Left: annotation is accepted if the left side matches;

● Right: annotation is accepted if the right side matches;

● Shared: annotation is accepted if the left or the right sides match;

● Overlap: annotation is accepted if there is any kind of match: exact, nested or intersected.

Such matching strategies allow a better understanding of annotation quality, since a non-exact matching does not mean that the correct concept was not recognized. For instance, considering gene and protein names, some systems and/or corpora include the organism name in the concept name and others do not, which remains a point of active discussion among expert annotators. Other point of disagreement is the inclusion of the tokens “protein” or “gene” as suffix or prefix, or including Greek letters in entity mentions [48]. Such analysis is also important since various post-NER tasks can be performed even if imprecise names are provided (e.g., relation and event mining).

The performance results on the various corpora were compared to previously published works to provide fair comparison. However, a complete comparison considering the five matching strategies is not always possible, since these different results are not stated in some works.

Regarding normalization and identifiers matching, we also consider two different matching strategies:

● Exact: annotation is accepted if one identifier is provided and it matches exactly with the gold standard;

● Contains: annotation is accepted if the provided list of identifiers contains the gold standard identifier.

Considering both matching strategies allows a more thorough analysis of the validity of the identifiers assigned to each entity mention. This evaluation was performed on the CRAFT corpus, since among the corpora considered in this work, only this one provides concept identifiers.

Common evaluation metrics are used to analyze and compare the achieved results: Precision (the ability of a system to present only relevant items); Recall (the ability of a system to present all relevant items); and F1-measure (the harmonic mean of precision and recall). These measures are formulated as follows:

$$P=\frac{\mathrm{TP}}{\mathrm{TP}+\mathrm{FP}},R=\frac{\mathrm{TP}}{\mathrm{TP}+\mathrm{FN}},F1=2\cdot \frac{P.R}{P+R}$$

where TP is the amount of true positives, FP the number of false positives and FN the amount of false negatives. Note that the presented results are micro-averaged, meaning that a general matrix of TP, FP and FN values is built from all documents to obtain final precision, recall and f-measure scores.

#### CRAFT

Considering the databases and ontologies used in the annotation of CRAFT, we defined six concept classes: species, cell, cellular component, chemical, gene and protein, and biological processes and molecular functions. Biological processes and molecular functions are grouped into a single class, since annotations are provided in a single file. Moreover, since gene and protein are provided through Entrez Gene (EZ) and Protein Ontology (PRO), we decided to perform two different evaluations regarding the recognition of named entities: *1*) against concepts provided by EZ; and *2*) against concepts provided by EZ and/or PRO. The performance on this NER task was compared against the results published by Verspoor et al. [27], who presented state-of-the-art results on CRAFT for sentence splitting, tokenization, POS tagging, syntactic parsing and named entity recognition. However, it only presents results for gene and protein recognition, where BANNER claims the best performing results using a ML model trained on the corpus of the BioCreative II gene mention corpus [10]. Thus, we decided to also use Cocoa and Whatizit to compare the achieved performance results. Since Cocoa concept classes do not match directly to the ones provided in CRAFT, we had to group them together to better fulfill the requirements and to achieve better results:

● Species: “Organism” and “Organism1”

● Cell: “Cell”

● Cellular Component: “Cellular component”, “Location” and “Complex”

● Chemical: “Chemical”

● Gene and Protein: “Protein”, “Molecule” and “Category”

● Biological Process and Molecular Function: “Bio Process” and “Process”

Whatizit was used through the “whatizitUkPmcAll” pipeline, which is used in Europe PubMed Central [49] to provide species, chemical, gene and protein, cellular component, biological process, molecular function and disorder concept annotations. To match the output with CRAFT, biological process and molecular function annotations were grouped into a single concept class, and disorder annotations were discarded.

**Figure 8:**
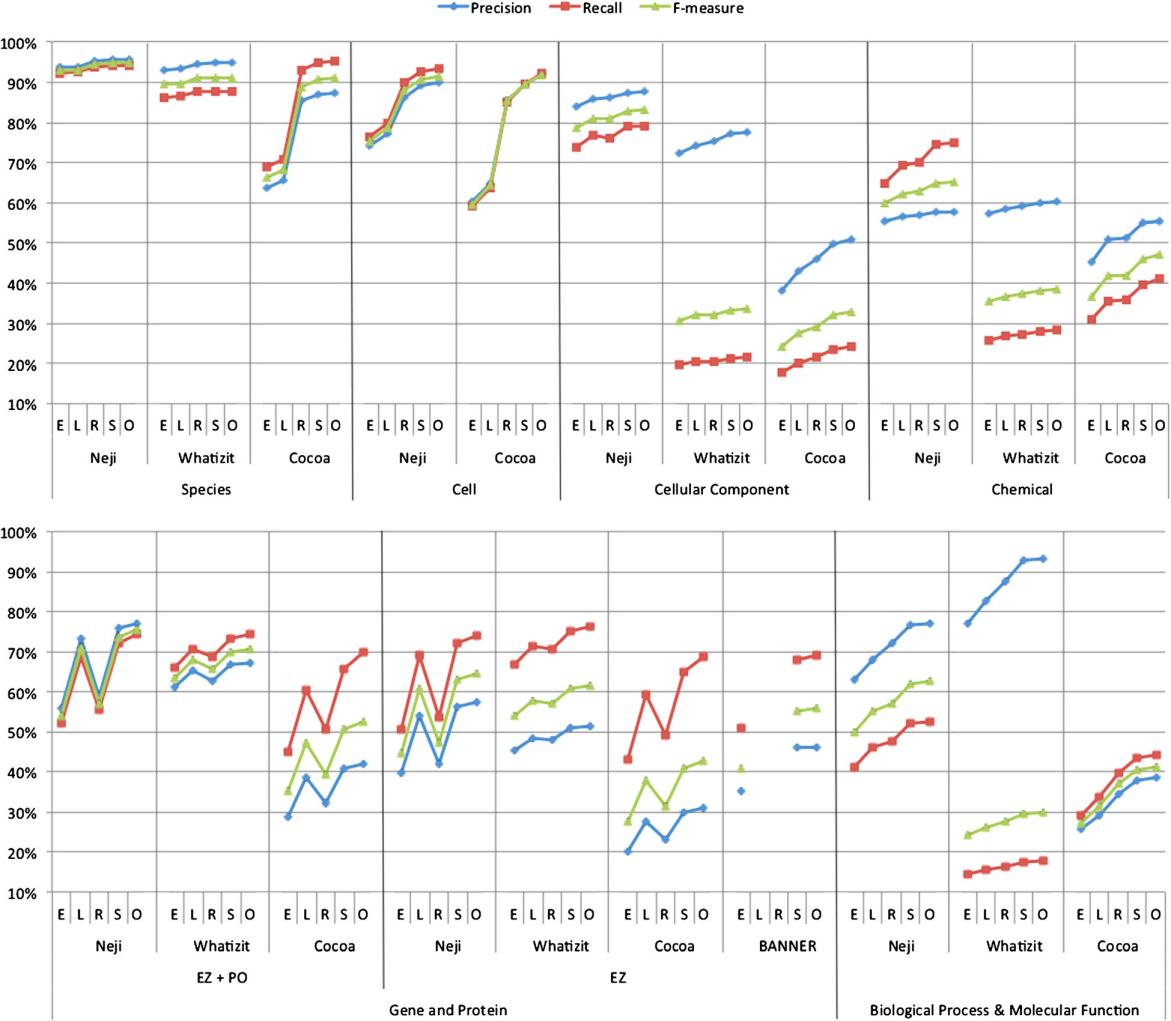
**Evaluation results for named entity recognition, considering precision, recall, and F1-measure achieved on CRAFT corpus, using exact (E), left (L), right (R), shared (S) and overlap (O) names matching.** Evaluation considers species, cell, cellular component, gene and protein, chemical, biological processes and molecular functions concept names.

Figure 8 presents the named entity recognition results achieved by Neji, Whatizit, Cocoa and BANNER on the CRAFT corpus, considering the various matching strategies. As we can see, there are considerable variations between the various matching strategies. For instance, on gene and protein names recognition, Neji, Whatizit and Cocoa perform much better on left matching in comparison to right matching, which confirms the previously referred variability of annotation guidelines, namely regarding the inclusion (or not) of word suffixes in concept names. Moreover, Neji and Cocoa also present better results on right matching on cell recognition, which indicates the presence of word prefixes on the gold standard that are being discarded by the automatic solutions. Those facts reflect the high variability of biomedical concept names, with different guidelines being followed by manual annotators and consequent generation of heterogeneous resources. Thus, as stated before, such discrepancies should be taken into account when evaluating solutions and corpora that follow different annotation guidelines.

Overall, Neji presents the best results, with significant improvements on various concept types, namely on concepts associated with GO (cellular component, biological process and molecular function), chemical and gene/protein. In more detail, we can see that Neji is the solution that presents overall best recall results without losing precision. Additionally, Neji also presents a positive constant behavior, with an average variation of 9% of F1-measure between exact and overlap matching. However, Whatizit is the most constant solution, with an average variation of 4% of F1-measure. On the other hand, Cocoa has the highest variation, with 18% of F1-measure.

Neji obtained state-of-the-art results on the recognition of species and cell concepts, with overlap F1-measure results of 94.7% and 91.5%, respectively. Extending LINNAEUS dictionaries allowed an improvement of more than 8% of F1-measure on overlap matching, from 86.1% to 94.7%. Nonetheless, both Cocoa and Whatizit present competitive results on species, and Cocoa also achieved state-of-the-art results on cell identification. Neji achieved an F1-measure of 83.2% on overlap matching in the recognition of cellular component names, which is significantly better than Cocoa and Whatizt. For instance, a detailed analysis showed that Cocoa’s performance is considerably degraded by the presence of terms such as “cell” and “cellular”. Regarding gene and protein recognition, Neji ML model presents better results than Cocoa, BANNER and Whatizit on left, shared and overlap matching. Its performance drop on exact and right matching appears to be a consequence of the different annotation guidelines in CRAFT and GENETAG, which was used to train Neji’s ML model. Specifically, species names, and suffixes such as “gene” and “protein” are considered as part of the concept name in GENETAG but not in CRAFT, causing an erroneous evaluation when exact matching is taken into account. Considering only the concepts from Entrez Gene, Neji has an improvement of more than 3% of F1-measure on overlap matching against the second best, Whatizit. When compared against BANNER, an improvement of 8% is achieved. Regarding Entrez Gene and/or Protein Ontology concepts, Neji presents an improvement of more than 5% of F1-measure against Whatizit and 23% against Cocoa, on overlap matching. Finally, the results achieved on chemical and biological processes and molecular functions are considerably better than Cocoa and Whatizit. However, we believe there is margin for progress, by: *1*) collecting more name variants to improve the recall for biological processes and molecular functions; and *2*) refining existing chemical dictionaries to improve precision.

Regarding normalization, previous works have presented performance evaluation results for specific entity types on specifically developed corpora, such as genes and proteins on AIMed [50] and/or BioInfer [51] corpora. Therefore, we evaluated the entity normalization performance achieved with Neji on the CRAFT corpus and compared it to the results obtained using the available pipelines in Whatizit, as this is the only freely available system that allows identification of various concept types with identifiers for each recognized concept name.

As presented previously, we combined various resources to collect as much names variants as possible, which results in identifiers from different resources for a single concept type. In some cases, both Neji and Whatizit use completely different resources than the ones used on CRAFT, such as in genes and proteins. Thus, in order to collect the performance results, we first converted the identifiers provided by Neji and Whatizit to the ones used in the CRAFT corpus. However, this mapping may deliver various problems, such as absent and ambiguous mapping, i.e., one identifier that is mapped to multiple identifiers, that will directly affect the obtained results. Table 1 presents a detailed analysis of the identifier mapping for Cell, Gene and Protein, and Biological Process and Molecular Function concept names, considering the annotations provided by Neji and Whatizit. Uniprot identifiers for genes and proteins were mapped to Entrez Gene (EZ) and Protein Ontology (PRO) identifiers using the mapping provided by Uniprot to EZ and the mapping provided by PRO to Uniprot. The UMLS concept identifiers assigned by Neji to Cell concept names were mapped to Cell Ontology (CL) identifiers through the mapping to the Foundational Model of Anatomy (FMA) ontology available in CL. However, this mapping is highly limited, since it only covers approximately 30% of CL. Finally, the dictionaries used in Neji for the recognition of Biological Process and Molecular Function concept names include some concepts from various UMLS semantic types that are not mapped to GO entries, as used in the CRAFT corpus.

**Table 1:** Number and percentage of identifiers and names mapped between different resources for cell, gene and protein, and biological process and molecular function concepts

	**From**	**To**	**Solution**	**# Identifiers**	**Mapped identifiers**	**# Concept names**	**Mapped concept names**
**Gene and protein**	Uniprot	Entrez Gene	Neji	51118	100%	13239	100%
			Whatizit	123136	53%	18079	95%
	Uniprot	Protein Ontology	Neji	51118	95%	13239	99%
			Whatizit	123136	22%	18079	78%
**Cell**	UMLS	Cell Ontology	Neji	8390	64%	5926	91%
Biological process and molecular function*	UMLS	Gene Ontology	Neji	6079	28%	5377	32%

*Only concept names with UMLS identifiers are considered.

The analysis of Table 1 shows that only 53% of the identifiers provided by Whatizit could be mapped to Entrez Gene. Nonetheless, most of the recognized concept names (95%) were associated to at least one identifier that could be mapped to an Entrez Gene identifier. On the other hand, all Uniprot identifiers provided by Neji were mapped to corresponding Entrez Gene entries. Considering the Uniprot to PRO mapping, only 22% of the identifiers provided by Whatizit were successfully mapped, while a PRO identifier could be assigned to 78% of the recognized concepts. Regarding Neji, 95% of the Uniprot IDs were mapped to PRO, and a PRO identifier was assigned to 99% of the recognized concepts. Various facts contribute to identifier mapping discrepancies between the two systems: 1) Neji uses Uniprot entries for 21 species while Whatizit uses the entire Uniprot database, resulting in more concept names and much more Uniprot identifiers; 2) the version of Uniprot used by Whatizit may not correspond to the version used for mapping; 3) not all Uniprot entries have a corresponding Entrez Gene entry; and 4) protein ontology does not map to all entries of Uniprot. Regarding cell identifiers mapping, 64% of the UMLS identifiers were successfully mapped into CL identifiers, resulting in 91% of the recognized concept names having CL identifiers. Finally, since Neji uses both GO and UMLS for representing Biological Process and Molecular Function concepts, we analyzed the mapping between the provided UMLS identifiers and corresponding GO entries. Considering only the annotations that contain UMLS identifiers, only 32% of the recognized concept names were mapped with GO identifiers. Overall, considering both UMLS and GO, 81% of the recognized concept names were provided with GO identifiers.

**Figure 9:**
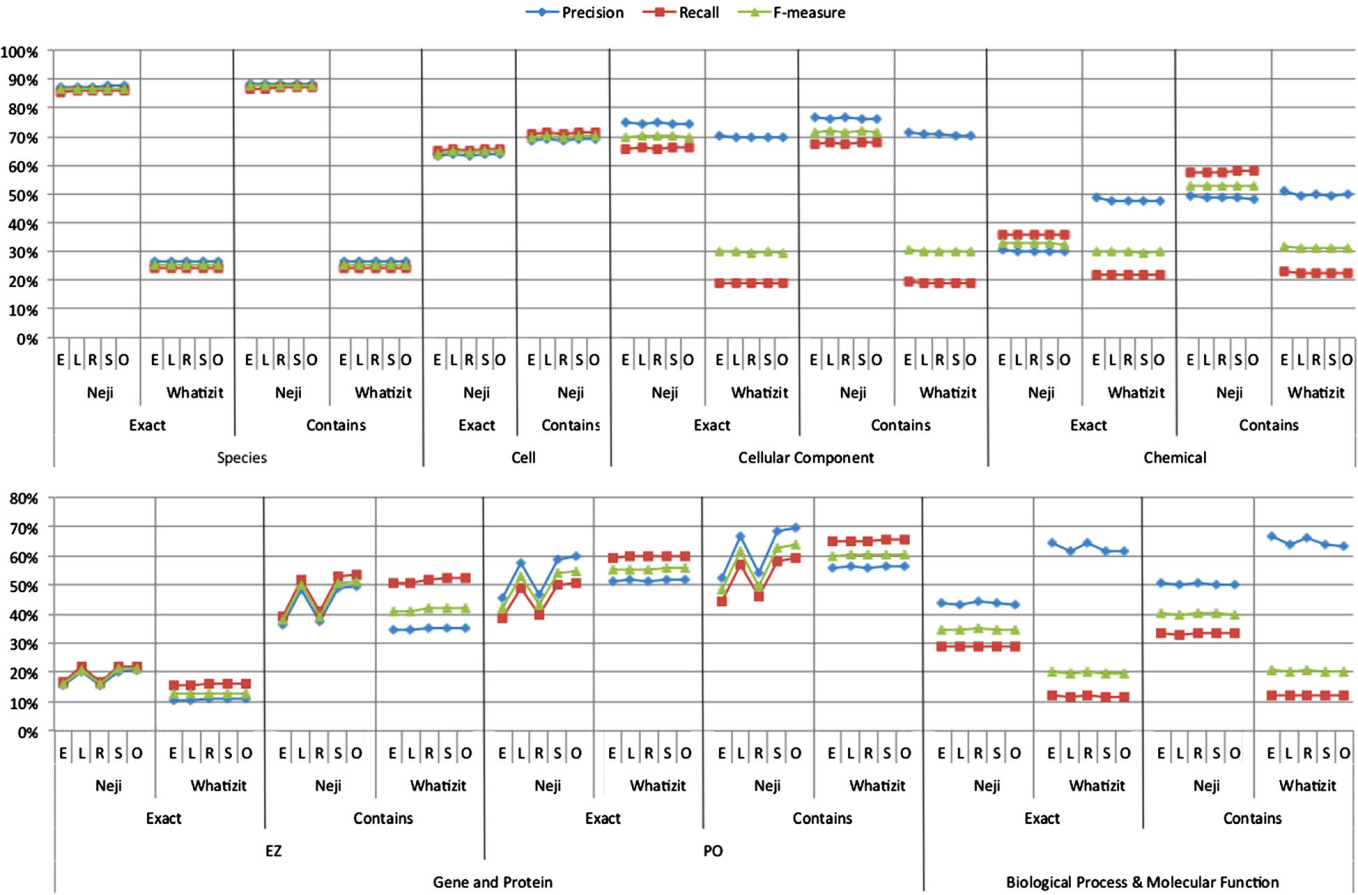
**Evaluation results for normalization considering precision, recall, and F1-measure achieved on CRAFT corpus, using exact (E), left (L), right (R), shared (S) and overlap (O) names matching and ‘exact’ and ‘contains’ matching of identifiers.** Evaluation considers species, cell, cellular component, gene and protein, chemical, biological processes and molecular functions concept names.

Figure 9 presents the normalization results achieved by Neji and Whatizit in the CRAFT corpus, after converting the identifiers as explained above, and considering the various strategies for matching the text chunks to the entries in the dictionary and the two identifier matching techniques (‘exact’ and ‘contains’). Overall, Neji considerably outperforms Whatizit on identifier matching for Species, Cellular Component, Chemical and Biological Process and Molecular Function concept names, with the exception of Gene and Protein concepts, where both solutions present similar results. Moreover, there is no high variability in identifiers matching when the various dictionary matching strategies are compared, again with the exception of gene and protein concept names. In this case, it is clear from the results that different annotation characteristics between the train and test corpora also have a substantial impact on the normalization performance. On the other hand, there is a significant difference in the results if we require that the correct identifier is returned (‘exact’) or that the correct identifier is included in the returned list of identifiers (‘contains’), highlighting the ambiguity in the concept names recognized in the texts.

Neji obtained state-of-the-art results in the recognition of species, with an F1-measure of 87.8% and no significant variance between ‘exact’ and ‘contains’ matching of identifiers. During the annotation of species in CRAFT, experts were required to assume the closest semantic match, which means that the mention “rat” was annotated as the genus “Rattus” (NCBI identifier 10114), even if from context it is known to be the common laboratory rat species “Rattus norvegicus” (NCBI identifier 10116). Such fact considerably affects the performance of Whatizit, since it only provides more specific species identifiers. For example, by considering just two of those cases and converting from “Rattus” (NCBI:10114) to “Rattus norvegicus” (NCBI:10116) and “Mus” (NCBI:10088) to “Mus musculus” (NCBI:10090), Whatizit results would achieve an F1-measure of 87.5%, similar to that achieved with Neji.

Neji presents competitive results on Cell concepts normalization, with a small variance between ‘exact’ (64.9% of F1-measure) and ‘contains’ identifier matching (70.5% of F1-measure). Such results represent a small drop when compared with the performance obtained on exact named entities matching (F1-measure of 75.4%). Regarding GO concept types, namely Cellular Component, Biological Process and Molecular Function, Neji considerably outperforms Whatizit, again with a small difference between ‘exact’ and ‘contains’ matching of identifiers. Considering ‘contains’ matching, Neji presents an F1-measure of 71.8% on Cellular Component, and 40.1% of F1-measure on Biological Process and Molecular Function. Comparing those results with exact named entity matching, they represent an average drop of 8% of F1-measure. The performance on Biological Process and Molecular Function is affected by the absent mappings between some UMLS concepts and GO identifiers.

Neji also outperforms Whatizit on Chemical concepts normalization, with an F1-measure of 33.1% on ‘exact’ and 53.1% on ‘contains’ identifier matching. The high difference between ‘exact’ and ‘contains’ matching reflects the high ambiguity present on ChEBI. For instance, the annotation “protein” on CRAFT contains the ChEBI identifier 36080 (“protein”), but the dictionary matching provides both 36080 and 16541 identifiers, which corresponds to “protein polypeptide chain” and also contains “protein” as a synonym. The best normalization results were achieved when exact named entity matching was considered, which shows that accepting left, right, shared and overlap matching may degrade normalization performance by leading to more false positives identifiers.

Finally, in order to present results for Gene and Protein concepts, two different evaluations were performed: 1) against Entrez Gene identifiers; and 2) against Protein Ontology identifiers. On both evaluations and systems, there is a considerable variation between the various names matching strategies and between ‘exact’ and ‘contains’ identifier matching, a consequence of the cross species ambiguity of gene and protein names. Regarding Entrez Gene, Neji and Whatizit present low performance results on ‘exact’ identifier matching, achieving F1-measures of 21.4% and 13.0%, respectively, when using overlap dictionary matching. When ‘contains’ identifier matching is considered, the performance of Neji and Whatizit improve considerably, achieving F1-measures of 52% and 42% for overlap dictionary matching, respectively. Concerning normalization to Protein Ontology, the achieved performance results are considerably better, with Neji and Whatizit achieving F1-measures of 55.0% and 55.6%, respectively, for ‘exact’ identifier matching and using overlap dictionary matching. When ‘contains’ matching is considered, both solutions present considerable improvements, with Neji achieving 64.0% of F1-measure and Whatizit 60.7%. Evaluating the normalization to both EZ and PRO, Whatizit presented the most constant behavior, a consequence of the different annotation guidelines followed in CRAFT and in the training corpus used to generate the ML model used by Neji. However, when all evaluation strategies are considered, Neji provides better results.

Overall, the presented analysis shows that Neji achieves competitive performance results on normalization, presenting small and anticipated performance drops when compared to named entities evaluation. Nonetheless, we consider that there is still margin for improvement, namely for chemicals and gene and protein normalization.

#### AnEM

To evaluate the recognition of anatomical concepts, we combined all sub-classes of the AnEM corpus into a single class. As a consequence, the systems are evaluated targeting the general ability to recognize anatomical entities, discarding the capability to classify and distinguish specific sub-anatomical classes. Thus, Neji is compared with the systems used in [41], i.e. MetaMap and NERSuite, which provide state-of-the-art results on this corpus. NERSuite was trained using the training part of the corpus, being optimized for these specific annotation guidelines. Cocoa provides anatomical classes following the AnEM classification approach. Thus, we annotated the corpus using Cocoa and mapped the respective classes to the single anatomical class. Body part concepts provided by Cocoa are also mapped to the single class.

**Figure 10:**
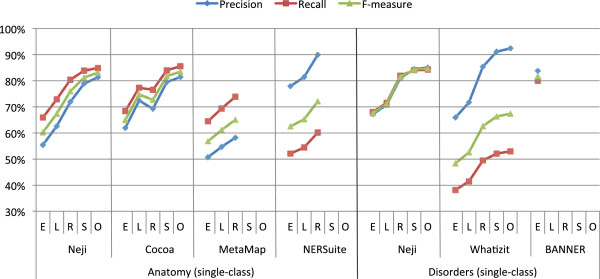
**Comparison of precision, recall, and F1-measure results achieved on AnEM and NCBI corpora for named entity recognition, considering exact (E), left (L), right (R), shared (S) and overlap (O) matching.** The various sub-classes from each corpus were merged into a single class, in order to evaluate the general ability to recognize disorder and anatomical concept names.

Figure 10 compares the results achieved by Neji, Cocoa, MetaMap and NERSuite on AnEM corpus, considering exact, left, right, shared and overlap names matching. Overall, there is a significant variation between the various matching techniques, which is observed in all systems. Even NERSuite has problems to identify the exact names’ boundaries, namely the right boundary. Such variation reflects the complexity of inferring the variable boundaries of anatomical names. Nonetheless, Cocoa is the system that presents better results, with 83.5% of F1-measure on overlap matching. Neji also presents competitive results, with 83.1% of F1-measure on overlap matching. On the other hand, MetaMap is the system that performs worst. Surprisingly, NERSuite does not perform better than Neji and Cocoa, which may indicate that ML-based solutions are not required for the general recognition of anatomical entities.

#### NCBI

Similarly to the AnEM corpus, we also combined NCBI sub-classes into a single class, in order to evaluate the general ability to identify names of disorders. The comparison is performed against BANNER and Whatizit. BANNER was used in [42] to present state-of-the-art results for ML-based solutions in this corpus. Although our approach is not ML-based and therefore not trained using the corpus, we believe this comparison is also relevant to provide feedback regarding the overall performance. Whatizit was used through the “whatizitDiseaseUMLSDict” pipeline.

Figure 10 compares the named entity recognition results achieved by Neji, Whatizit and BANNER on the NCBI corpus. There is also a significant variation between the various matching techniques, namely on right matching. This means that various concepts are not precisely identified due to the presence or absence of word prefixes. For instance, in our case, the gold standard annotation “atrophic benign epidermolysis bullosa” is typically provided just as “epidermolysis bullosa”. Even though the text chunk is not correct, it points to a related concept. Comparing the two dictionary-based approaches, Neji presents significantly better results than Whatizit, with an improvement of more than 17% of F1-measure on overlap matching. On the other hand, BANNER, a ML-based solution trained on this corpus, achieved significantly better results than Neji when exact matching is considered. However, the high-performance results obtained with Neji when fuzzy matching is used, seem to indicate a mismatch between the terms in the dictionary used and the annotations on this corpus, which may be a consequence of two factors:

● High variability found on more specific concept names and consequently their absence in the dictionary. For instance, the gold mention “attenuated adenomatous polyposis coli” is annotated by Neji as “adenomatous polyposis coli”, which are two related but different concepts, since the gold mention is more specific;

● Inconsistencies in following the annotation guidelines. For instance, the gold mentions “breast/ovarian cancer” or “breast and ovarian cancer” are annotated as a single concept name. However, UMLS does not contain such terms, since they point to two different UMLS concepts.

Summarizing, we can argue that Neji presents highly competitive results, with significant improvements for some semantic groups, namely species, cell, cellular component, gene and protein, and anatomy.

### Speed evaluation

One important characteristic of concept recognition solutions is annotation speed, since large data sets may be annotated to collect as much information as possible. To evaluate the annotation speed achievable with Neji, various experiments were performed using the CRAFT corpus, which contains 67 full-text articles with 21749 sentences. The documents were processed on a machine with 8 processing cores @ 2.67 GHz and 16GB of RAM.

The annotation process using the dictionaries and ML model previously described and using 5 threads took 124 seconds, which corresponds to processing 175 sentences per second. Thus, it took 1.8 seconds on average to process a full text article. Considering that MEDLINE contains 11 millions abstracts [52], and that each abstract contains on average 7.2 sentences [53], this configuration could annotate the entire MEDLINE in five days. Since generating the complex features for the ML model and collecting POS and chunking features is resource intensive, we also measured the processing speed without using ML, applying only dictionary matching and tokenization from the NLP module. With this configuration, the CRAFT corpus was processed in 18 seconds, which corresponds to 1208 sentences/second. Thus, a full text article was processed in 0.28 seconds, and the entire MEDLINE could predictably be annotated in 18 hours. To contextualize the achieved results, we compared Neji with other existing tools. Even though BANNER applies ML for gene and protein names recognition only, it took more than 9 minutes to annotate CRAFT. On the other hand, the rule-based solution MetaMap took more than 2 minutes to process a single full-text file. We believe that the presented processing speeds provide a positive contribution to the biomedical community, making annotation of large data sets with dozens of biomedical concepts easily accessible.

## Discussion

The inherent characteristics, features and performance provided by the Neji framework represent various technical and theoretical advantages to end-users, contributing to an improved and faster research in biomedical text mining and information extraction. First of all, the large dictionaries used in our experiments, in combination with the achieved processing speeds, are good indicators of the scalability of the presented solution. Additionally, the achieved high-performance results against gold standard corpora show the solution’s reliability. Overall, the flexibility, scalability, speed and performance results offered by the proposed framework expedite the processing of the increasing scientific biomedical literature. The features provided greatly simplify NER and normalization tasks, offering annotations for a large number of entity types using both dictionary and machine learning-based approaches. Using the state-of-the-art modules incorporated in Neji, developers and researchers can bypass normally complex and time-consuming tasks, allowing them to focus on further analysis of these annotations. Users can also take advantage of the integrated natural language processing tools, eliminating the need for developing wrappers or integration solutions. The adoption of the same techniques for linguistic processing means that all modules are based on the same consistent information, such as tokens, lemmas, POS tags, chunks and parsing trees. This approach builds an integrated development ecosystem that minimizes cascading errors. For instance, if concept recognition is performed using linguistic information from one parser, and relation extraction is performed afterwards using information provided by another parser, it is hard to keep consistency between the two solutions, since the application of distinct sentence splitting and tokenization techniques provide different and hard to combine interpretations of data. Thus, performing all tasks using the same linguistic information will deliver better and more consistent results.

Besides using the provided modules directly, researchers may also adapt them or integrate new ones, allowing the construction of specialized processing pipelines for text mining purposes. As presented, Neji is ready to be used by users with different levels of expertise. It allows obtaining heterogeneous concepts of several types in a straightforward way, by using the CLI tool or by building a pipeline with existing modules. Users also have the power to optimize concept recognition for their specific goals, which is achieved by having access to the innovative concept tree. Such structure supports both nested and intersected annotations and, combined with the support for multiple identifiers from different semantic groups per concept, enables easy detection of ambiguity problems. Additionally, Neji also integrates helpers for simple concept disambiguation, merging of nested annotations and selection intersections. If required, users can also develop their own modules, such as readers, writers or WSD. Overall, Neji was built considering different development configurations and environments: *a*) as the core framework to support all developed tasks; *b*) as an API to integrate in your favorite development framework; and *c*) as a concept recognizer, storing the results in an external resource, and then using your favorite framework for subsequent tasks.

A large and diverse set of annotations can be obtained by processing a large set of documents. Such annotations can be exploited in various ways. Perhaps, the most straightforward one is to use these annotations together with the provided identifiers and connections to ontologies and other domain resources, to support a semantically enabled literature retrieval system [54-56]. Using these annotations, it also becomes simpler to implement a query expansion scheme [57], taking advantage of the ontological relationships between the identified concepts. Another use of such annotations is to extract co-occurrence based association metrics between concepts [58,59]. This can also be extended to extracting semantic concept profiles that represent the semantic context in which a given concept occurs, as described in [60]. Creating these profiles is highly dependent on the annotation of a large set of documents with diverse and rich concepts from various semantic types. Co-occurrence and context-based association metrics can in turn be exploited for discovering implicit (A-B-C) concept relations from the literature, therefore supporting hypothesis generation and knowledge discovery.

With this analysis, we show that Neji is a good starting point to develop complex biomedical text mining projects, supporting advanced and reliable features and giving users the power to choose the best behaviors considering the complete tree of recognized concepts and their specific goals.

## Conclusion

This article presents Neji, an open source and modular framework optimized for general biomedical concept recognition. It was developed considering scalability, flexibility, speed and usability. Neji integrates state-of-the-art and optimized solutions for biomedical natural language processing, such as sentence splitting, tokenization, lemmatization, POS tagging, chunking and dependency parsing. Concept recognition is supported through dictionary matching and machine learning, integrating features to perform normalization of recognized chunks of text. Various known biomedical input and output formats are also supported, namely Raw, XML, A1 and CoNLL. Recognized concepts are stored in an innovative concept tree, supporting nested and intersected concepts with multiple identifiers. Such structure provides enriched concept information and gives users the power to decide the best behavior for their specific goals, using the included methods for handling and processing the tree.

We also evaluated Neji against a wide variety of biomedical entity types, achieving high-performance results on manually annotated corpora. To the best of our knowledge, the analysis presented constitutes the most comprehensive evaluation of named entity recognition and normalization for such a heterogeneous set of biomedical concept types. Additionally, the presented processing speeds make the annotation of large document sets a reality. We also described the simple usage of Neji through the integrated CLI tool, which allows annotating thousands or millions of documents with a simple bash command. Furthermore, we illustrated the simplicity of developing a custom pipeline using existing modules.

We believe that the characteristics and complex features provided by Neji fill the gap between general frameworks (e.g., UIMA and GATE) and more specialized tools (e.g., NER and normalization). It streamlines and facilitates biomedical concept recognition, using both dictionary and machine learning-based approaches to extract multiple concept types in an integrated ecosystem. Neji simplifies concept recognition tasks in biomedical information extraction, and it can be easily integrated in complex workflows contributing towards more accurate knowledge discovery.

There are already various solutions developed and/or being developed on top of Neji, such as: *a*) a solution to extract gene-drug relations from scientific articles; *b*) a web-based solution and respective web-services for on-demand biomedical concept recognition [61]; *c*) an information retrieval solution for knowledge discovery focused on degenerative diseases; and *d*) an information retrieval system focused on relevant and informative sentences.

## Availability and requirements

**Project name:** Neji

**Project home page:** http://bioinformatics.ua.pt/neji

**Operating system(s):** Platform independent

**Programming language:** Java

**Other requirements:** Java 1.7 or higher

**License:** Creative Commons Attribution-NonCommercial-ShareAlike 3.0 Unported License

**Any restrictions to use by non-academics:** Non-commercial use

## Endnotes

^a^A. thaliana, B. taurus, C. elegans, C. reinhardtii, D. rerio, D. discoideum, A. mellifera, C. albicans, D. melanogaster, H. sapiens, M. musculus, R. norvegicus, S. cerevisiae, Hepatitis C virus, M. pneumoniae, P. falciparum, P. carinii, S. pombe, Z. mays, E. coli and X. laevis.

## Competing interests

The authors declare that they have no competing interests.

## Authors’ contributions

DC participated in the design and implementation of the framework and drafted the manuscript. SM and JLO conceived the study, participated in its design and coordination and helped to draft the manuscript. All authors read and approved the final manuscript.
